# Functional specialization of Aurora kinase homologs during oogenic meiosis in the tunicate *Oikopleura dioica*

**DOI:** 10.3389/fcell.2023.1323378

**Published:** 2023-12-07

**Authors:** Haiyang Feng, Eric M. Thompson

**Affiliations:** ^1^ Institute of Biological Sciences, Jinzhou Medical University, Jinzhou, China; ^2^ Sars International Centre for Marine Molecular Biology, University of Bergen, Bergen, Norway; ^3^ Department of Biological Sciences, University of Bergen, Bergen, Norway

**Keywords:** tunicate, Aurora kinase, INCENP, interaction/scaffolding proteins, oogenic meiosis, molecular evolution

## Abstract

A single Aurora kinase found in non-vertebrate deuterostomes is assumed to represent the ancestor of vertebrate Auroras A/B/C. However, the tunicate *Oikopleura dioica*, a member of the sister group to vertebrates, possesses two Aurora kinases (Aurora1 and Aurora2) that are expressed in proliferative cells and reproductive organs. Previously, we have shown that Aurora kinases relocate from organizing centers to meiotic nuclei and were enriched on centromeric regions as meiosis proceeds to metaphase I. Here, we assessed their respective functions in oogenic meiosis using dsRNA interferences. We found that Aurora1 (Aur1) was involved in meiotic spindle organization and chromosome congression, probably through the regulation of microtubule dynamics, whereas Aurora2 (Aur2) was crucial for chromosome condensation and meiotic spindle assembly. *In vitro* kinase assays showed that Aur1 and Aur2 had comparable levels of kinase activities. Using yeast two-hybrid library screening, we identified a few novel interaction proteins for Aur1, including c-Jun-amino-terminal kinase-interacting protein 4, cohesin loader Scc2, and mitochondrial carrier homolog 2, suggesting that Aur1 may have an altered interaction network and participate in the regulation of microtubule motors and cohesin complexes in *O. dioica*.

## Introduction

The Aurora kinases are a group of evolutionarily conserved serine/threonine protein kinases that regulate diverse cellular events during mitosis and meiosis (Hochegger et al., 2013). Aurora originated as a single gene in the last eukaryotic common ancestor (LECA) and evolved independently in plants, fungi, protists, and metazoans (Brown et al., 2004). However, the Aurora kinase sequences of invertebrates are highly divergent from those of vertebrates and often exhibit long-branch attraction artifacts in phylogenetic analyses (Brown et al., 2004). The single, bifunctional Aurora present in the echinoderm starfish and tunicate ascidians are believed to represent the ancestral kinase that gave rise to Auroras A and B in vertebrates (Abe et al., 2010; Hebras and McDougall, 2012). Whether this scenario is generally applicable in chordates requires stringent functional evidence across closely related phyla, in order to better understand the selection pressures that restrain the concerted functions of Aurora homologs.

In species with a single Aurora, it functions at the spindle poles, chromosomes, and central spindle in mitosis (Li et al., 2008; Abe et al., 2010; Hebras and McDougall, 2012). In metazoans possessing two Aurora kinases, functional studies consistently reveal the division of labor between A- and B-type Aurora kinases at the spindle poles and equator (Hochegger et al., 2013). In contrast, reports on the meiotic functions of Aurora kinases in a few model organisms are sparse and less consistent (Nguyen and Schindler, 2017). Aurora A has a common role in regulating microtubule assembly and maintenance in the meiotic spindle, but this task is carried out at different sites. Aurora A associates with acentriolar microtubule organizing centers (aMTOCs) at the spindle poles in mouse oocytes and induces the fragmentation of aMTOCs through the phosphorylation of polo-like kinase 1 (PLK1) (Blengini et al., 2021), while active Aurora A/AIR-1 is concentrated on chromosomes during prometaphase I and on interchromosomal microtubules during anaphase I in *C. elegans* oocytes (Sumiyoshi et al., 2015). The chromosomal passenger complex (CPC) kinase, Aurora B, is involved in multiple aspects of meiotic division, such as chromosome condensation, cohesion, and error correction in kinetochore-microtubule (KT-MT) attachments. In *C. elegans* oocytes, Aurora B/AIR-2 is localized on the equatorial axis of holocentric bivalents where it provides spatial cues to load condensin I, promotes ring complex (RC) assembly, and releases interhomolog cohesins at the onset of anaphase I (Rogers et al., 2002; Collette et al., 2011; Divekar et al., 2021). In *Drosophila* oocytes, Aurora B/*ial* interacts with non-centromeric chromosomes and regulates spindle bipolarity and chromosome biorientation at the central spindle ring (Radford et al., 2012). In mouse oocytes, Aurora C replaces Aurora B at the interchromatid axis (ICA) and centromeres and also localizes on aMTOCs. Aurora C regulates the loading of condensin, chromosome alignment, correction of erroneous KT-MT attachments, and aMTOC clustering (Balboula and Schindler, 2014; Nguyen et al., 2014; Balboula et al., 2016). Subtle differences in the localizations and catalytic activities of Aurora kinases are imposed by their interacting proteins (Carmena et al., 2009). It remains unclear to what extent the distinct requirements of Aurora kinases in meiotic progression reflect the diversity of their interaction proteins and substrates in different organisms. This is of interest in the context of gene gains and losses during metazoan evolution.

The tunicate, *Oikopleura dioica*, in the sister group to vertebrates, is an emerging model used to study variants in cell cycle regulation (Delsuc et al., 2006; Campsteijn et al., 2012; Ma et al., 2022). It has a compact genome of 70 Mb, accompanied by massive gene losses and lineage-specific gene duplications (Denoeud et al., 2010; Ferrández-Roldán et al., 2021). In contrast to ascidians, appendicularians possess two Aurora kinase homologs. In this study, we assessed the distinct roles of two Aurora kinases during oogenesis in *O. dioica*. We found that Aurora1 regulated microtubule dynamics during meiotic spindle assembly, and Aurora2 was essential for chromosome condensation and meiotic spindle assembly. We also identified some novel interaction proteins for Aurora1, suggesting new avenues for further investigation of the evolutionary trajectory of Aurora kinases in chordates.

## Materials and methods

### Animal culture and microinjection

*Oikopleura dioica* culture was carried out as before (Bouquet et al., 2009). dsRNA was synthesized using the T7 RiboMAX™ Express RNAi System (Promega) from a 300–400-bp DNA fragment within the CDS of the target gene as templates. At day 4, females were placed in artificial seawater, removed from their houses, anesthetized in ethyl 3-aminobenzoate methanesulfonate salt (MS-222, 0.125 mg/mL, Sigma), and injected with 400 ng/μL of dsRNA solutions. Shortly before spawning, the injected mature females were individually transferred to artificial seawater in six-well plates coated with 0.1% gelatin. After spawning, oocytes were collected for RT–qPCR or used for phenotypic analyses. Capped Aur1-GFP mRNA was synthesized from a linearized pANZ-OdAur1-GFP fusion construct (IDT) using the mMESSAGE mMACHINE T7 Transcription Kit (Ambion) and was injected into the 1-cell embryos at 400 ng/μL. The injected embryos were collected at 2–8 cell stages for immunostaining using an anti-GFP antibody (AMS Biotechnology, TP401) to detect the localizations of Aurora1.

### Antibodies

Primary antibodies include anti-Histone H3-pS10 (Upstate, 06–570), anti-H3-pS28 (Abcam, ab10543), anti-Aurora A (pT288)/Aurora B (pT232)/Aurora C (pT198) (Cell Signaling Technology, D13A11), anti-α-Tubulin (Abcam, ab6161), anti-GFP (AMS Biotechnology, TP401), and anti-His tag (Santa Cruz, sc-8036). Secondary antibodies against rabbit, mouse, or rat IgG (conjugated Alexa Fluor 488, 568 or HRP) were supplied by molecular probes. Primary antibodies were used at 1:100 dilutions, and secondary antibodies were used at 1:300 dilutions for immunofluorescence. Primary and secondary antibodies were used at 1:2000 and 1:5000 dilutions, respectively, in Western blots.

### Imaging

Following immunostaining, nuclei were counterstained with 1 µM TO-PRO-3 iodide (Molecular Probes). Fluorescence images were acquired using a Leica SP5 Confocal with 40x/NA1.25 objective. Brightfield images were acquired using a Nikon TE2000-S microscope with 20x/NA0.45 objective. All images were processed using ImageJ.

### Protein expression and purification

The coding sequences of OdAur1 or OdAur2 and INbox sequences (residues 493–572) of OdINCENPa were cloned into the first and second cassettes of a bi-cistronic vector pHTvAMP1-SGC (gift from Dr. Jonathan M. Elkins) (Abdul Azeez et al., 2019). The resulting constructs expressed as the complexes of his-tagged Aurora (N-terminal fusion residues: MGSSHHHHHHSQDPENLYFQGANSSSARLQ for OdAur1 and MGSSHHHHHHSQDPENLYFQGANS for OdAur2) and INboxes with an additional N-terminal methionine. The constructs were transformed into *E. coli* BL21 (DE3)-R3-pRARE2. The resulting colonies were inoculated into the 5-mL SOB media and cultured overnight. These overnight cultures were inoculated into the 1-L SOB media and were grown to OD600 of 0.5 at 37°C. A final concentration of 0.5 mM IPTG was added to induce protein expression, and afterward, the cultures were grown at 20°C overnight. The next day, the bacterial cells were harvested by centrifugation, resuspended in binding buffer (50 mM Tris-HCl pH 7.5, 500 mM NaCl, 20 mM imidazole, 5% glycerol, and protease inhibitor cocktail), and lysed by sonication. After high-speed centrifugation, the supernatant was recovered and passed through a 0.45-µm syringe filter before loading to a column packed with 1 mL of Ni Sepharose resin (GE Healthcare). The resin was washed with an increasing gradient (20 mM–100 mM) of imidazole. The purified proteins were eluted with three bed volumes of 250 mM imidazole and concentrated using an Amicon Ultra-4 10K centrifugal filter device (Millipore). Protein concentrations were determined by the Bradford assay (Thermo Fisher).

### Aurora kinase assays

Equal amounts of purified proteins were used in kinase assays with the CycLex Aurora Family Kinase Assay Kit (MBL|Medical & Biological Laboratories), following the manufacturer’s instructions. In brief, 10 µL of protein sample and 90 µL of kinase reaction buffer containing 0.125 mM ATP were added to the well that was pre-coated with “Aurora-substrate-1” and were incubated for 30 min at 30°C. After washing the well, 100 µL of HRP-conjugated antibody, recognizing the phosphorylated substrate, was added and incubated for 30 min at 25°C. After another round of washing, 100 µL of chromogenic reagent was added and incubated for 15 min at 25°C. Finally, 100 µL of stop solution was added to terminate the color development reaction. Absorbance was measured at 450 nm with a TECAN microplate reader.

### Yeast two-hybrid library screening

Total RNA from mixed samples containing oocytes and early embryos was extracted using the RNAqueous™-Micro Total RNA Isolation Kit (Ambion). A cDNA library was generated and incorporated into the prey vector pGADT7-pla using the all-direct method (Shanghai Biogen Biotechnology Corporation) and then transformed into a yeast strain Y187 following the LiAc transformation procedure (Clontech) and, hereafter, named the prey library as pGADT7-Od-pla. A bait vector pGBKCg containing Aur1 (pGBKCg-Aur1) was transformed into AH109. The autoactivation of the bait vector was tested and confirmed that the bait vector did not activate *HIS3*, *ADE2*, and *LacZ* reporters. Next, prey library cells were incubated with the overnight culture of bait cells for 24 h at 30°C. One aliquot of zygotes was gradient-diluted and plated on SD-TL plates to calculate mating efficiency. The rest were plated on SD-TLH +5 mM 3AT plates and maintained at 30°C for 3–4 days. Positive colonies were further inoculated on SD-TLHA plates to test the *ADE2* reporter. Plasmids were rescued from the colonies that passed both *HIS3* and *ADE2* tests and sequenced. Sequences were blasted against NCBI and OikoBase (http://oikoarrays.biology.uiowa.edu/Oiko/) databases to retrieve their protein identities and mRNA expression profiles at different developmental stages of *O. dioica*.

## Results

### Identification and functional characterization of Aurora homologs in appendicularians

**FIGURE 1:**
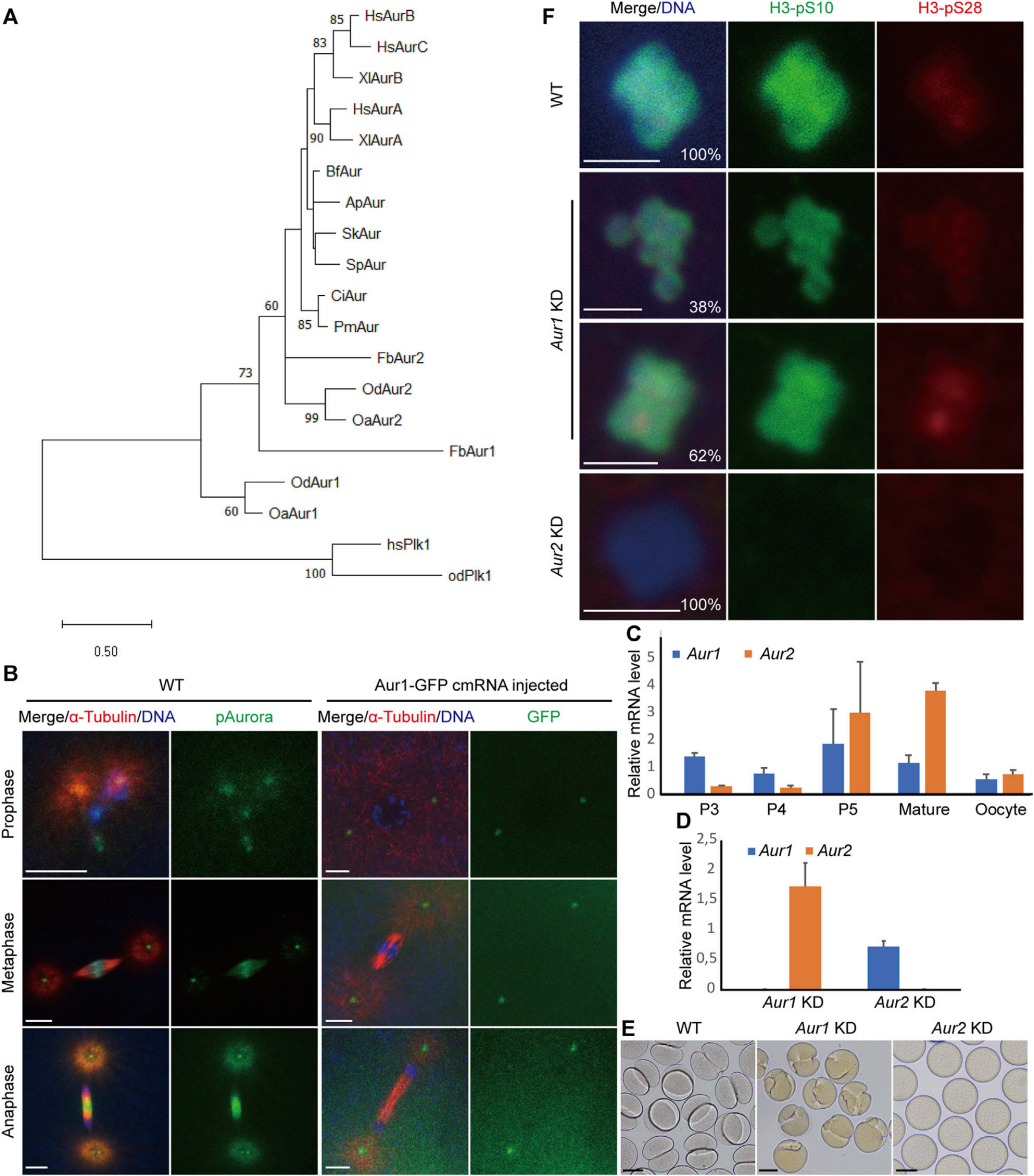
Identification, expression profiles, and distinct functions of Aurora1 and Aurora2 kinases during oogenic meiosis in *Oikopleura dioica*. **(A)** Phylogenetic relationships of Aurora kinases of three larvaceans in deuterostomes. The tree was constructed in MEGA11 using the maximum likelihood method based on the alignment of the catalytic domains of Aurora kinases (Supplementary Data S1) (Tamura et al., 2021). An LG + G amino acid substitution model was used in a 1,000-iteration bootstrap test. Only bootstrap percentages >50% are shown on the nodes. Tree was drawn to scale, with branch lengths measured in the expected number of substitutions per site. Plk1 kinases were used as an outgroup to root the tree. The abbreviations of the selected species are as follows: Hs, *Homo sapiens*; Xl, *Xenopus laevis*; Od, *Oikopleura dioica*; Oa, *Oikopleura albicans*; Fb, *Fritillaria borealis*; Ci, *Ciona intestinalis*; Pm, *Phallusia mammillata*; Bf, *Branchiostoma floridae*; Sk, *Saccoglossus kowalevskii*; Ap, *Asterina pectinifera*; and Sp, *Strongylocentrotus purpuratus*. **(B)** Localizations of combined Aurora signals and Aurora1 during embryonic mitoses. Scale bars: 5 µm. **(C)** Relative mRNA levels of *Aur1* and *Aur2* during *O. dioica* oogenesis. Coenocytic oogenesis is subdivided into five phases, P1 to P5 (Ganot et al., 2007). P3 corresponds to pachytene and diplotene, during which the coenocyst grows rapidly. In P4, a subset of pro-oocytes increases in size by cytoplasmic transfer through ring canals and meiotic chromatin condenses. During P5, oocytes attained their full size, and nurse and excess meiotic nuclei underwent apoptosis. Subsequently, oocyte maturation is completed, and spawning occurs *via* the rupture of the gonad wall. Mean values normalized to EF1 transcripts are shown with standard error bars (n = 3). **(D)** Knockdown efficiencies of target genes in spawned oocytes were obtained using RT–qPCR after dsRNA-mediated knockdowns of *Aur1* and *Aur2*, respectively. Fold changes of mRNA levels relative to those in wild type are shown with standard error bars (n = 3). **(E)** At 30 min after exposure to wild-type sperm, WT embryos underwent first cleavage (left). *Aur1* KD embryos showed “boxing glove”-like shapes (middle), whereas *Aur2* KD oocytes were non-viable (right). Scale bars: 50 µm. **(F)** Knockdowns of *Aur1* and *Aur2* had distinct effects on histone H3-S10 and H3-S28 phosphorylation on oocyte chromosomes. In WT (top), the H3-pS10 signal was present along the entire chromosomes, and H3-pS28 was enriched at centromeres. In *Aur1* KD (n = 52, middle), although H3-pS10 appeared normal, 38% of oocytes showed faint signals of H3-pS28 spreading along the entire chromosomes, and 62% of oocytes showed enriched signals of H3-pS28 at centromeres. In all *Aur2* KD oocytes (n = 36, bottom), both H3-pS10 and H3-pS28 signals were absent on decondensed chromosomes. Scale bars: 2 µm.

Appendicularians, also known as larvaceans, include three families: Oikopleuridae, Fritillaridae, and Kowalevskiidae. In the available genome and transcriptome databases of appendicularians, we ran a local tBLASTn search using Aurora ortholog sequences from both invertebrates and vertebrates as queries. We consistently found two Aurora homologs with very significant E-values (2e-066–7e-144) in *Oikopleura dioica*, *Oikopleura albicans*, and *Fritillaria borealis*. Among the vertebrates, phylogenetic trees clearly showed that vertebrate Aurora A formed one orthologous clade. Auroras B and C in mammals and Aurora B in amphibians formed another clade. However, the nodes leading to other invertebrate deuterostomes were not well resolved with high-bootstrap values. The single Aurora observed in amphioxus, hemichordates, and echinoderms was weakly clustered together. Auroras in ascidians have occupied a separate branch. Auroras in appendicularians underwent rapid evolution as witnessed by their long-branch lengths, and they also underwent an independent gene duplication event which generated two lineages: Aurora1 and Aurora2 in *O. dioica*, *O. albicans*, and *F. borealis* (Figure 1A; Supplementary Data S1). OdAur1-capped mRNA with a C-terminal GFP fusion was injected into 1-cell embryos, and the localizations of OdAur1-GFP fusion protein during early embryonic mitoses were analyzed. Aurora1 was localized on centrosomes throughout mitosis. Using a commercial phospho-Aurora antibody that can recognize the phosphorylated forms of both Aurora1 and Aurora2 in *O. dioica*, we assessed the combined signals of the two Aurora kinases. As expected, during early embryonic cleavages, signals were found on centrosomes and chromosomes in prophase and metaphase, and on centrosomes and the central spindle in anaphase (Figure 1B). As such, Aurora2 would occupy the chromosome-central spindle axis. This suggests that Aurora1 and Aurora2 in *O. dioica* represent the polar and equatorial forms of Aurora kinases, respectively, and they are most likely to be functional equivalents of vertebrate Auroras A and B during mitosis.

The expression profiles of the two Aurora kinases during oogenesis were analyzed by RT–qPCR. Their transcripts co-existed during oogenesis, with maximum expression levels at P5 and maturation phases during which nurse nuclei exported RNAs and proteins to rapidly growing pro-oocytes (Figure 1C). Our previous studies have shown that combined Aurora signals were present in meiotic nuclei and organizing centers (OCs) during P3, relocated to selected meiotic nuclei during P4, and enriched toward centromeric regions during prometaphase I. This coincided with the changes of histone H3-S10 and H3-S28 phosphorylation from P3 to P4 in selected meiotic nuclei and acentrosomal spindle assembly during prometaphase I (Øvrebø et al., 2015; Feng and Thompson, 2018).

To explore their potential roles in the aforementioned meiotic features, dsRNA knockdowns (*Aur1* KD or *Aur2* KD) were carried out by gonad injection. Knockdown efficiencies were calculated by RT–qPCR, showing that the mRNAs of target genes dropped to extremely low levels, whereas paralogous genes were not significantly affected (Figure 1D). After exposure to wild-type sperm, some *Aur1* KD oocytes seemed to extrude two polar bodies with normal timing. However, when the first mitotic division started, cleavage furrows emerged from one side of most zygotes and abnormally progressed inwards, appearing as “boxing gloves” at 30 min post-fertilization (Figure 1E). Thereafter, some furrows regressed, whereas others formed one or more furrows from other locations, resulting in two or three cells with unequal sizes or disrupted division axes (Supplementary Figure S1). In contrast, *Aur2* KD oocytes were non-viable, and polar body extrusion was not observed (Figure 1E). We suspect that defective mitotic cleavages in *Aur1* KD might be caused by defects in meiotic progression. We have previously shown that H3-S10 and H3-S28 phosphorylation can indicate the progression of meiosis I. Therefore, we assessed these two histone modifications in *Aur1* KD and *Aur2* KD oocytes (Figure 1F). H3-pS10 was present on the entire chromosomes in all the *Aur1* KD oocytes that have been analyzed, similar to WT, but H3-pS28 was either spread along entire chromosomes in 38% of oocytes or enriched on centromeres in 62% of oocytes. This suggests that Aurora1 is involved in promoting the progression of prometaphase I. In all the *Aur2* KD oocytes, both H3-pS10 and H3-pS28 were absent, and chromosomes were decondensed, reminiscent of *INCENPa* knockdown phenotypes (Feng et al., 2019). This confirms that Aurora2 and INCENPa, as the kinase and scaffold proteins, respectively, in the CPC, are essential for H3-S10 and -S28 phosphorylation and chromosome condensation during oogenic meiosis of *O. dioica*.

### Distinct roles of Aurora1 and Aurora2 in regulating acentrosomal spindle assembly

**FIGURE 2:**
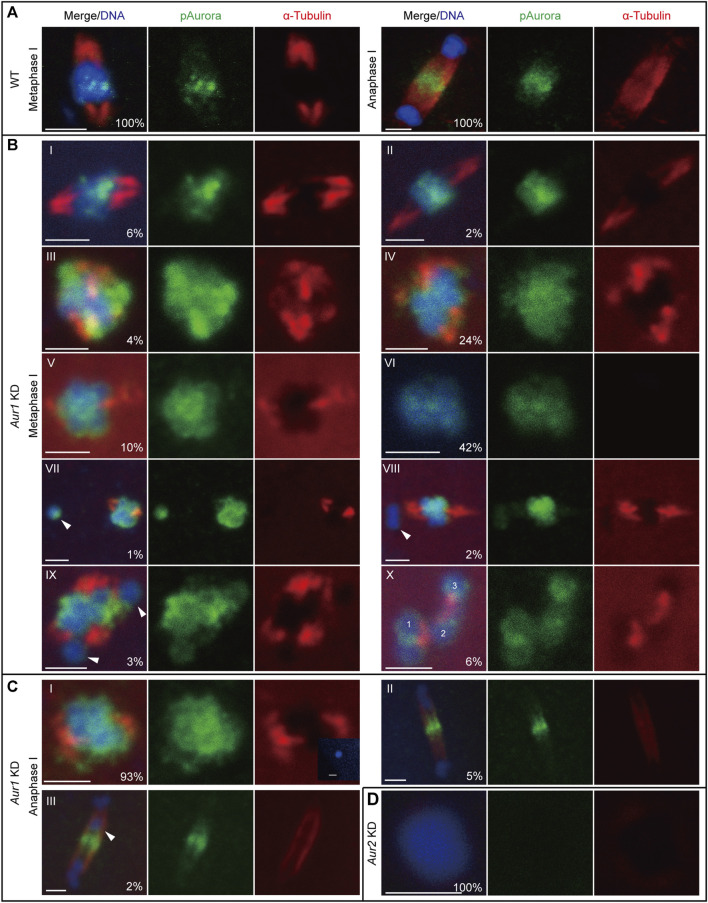
Differential requirements of Aurora1 and Aurora2 in meiotic spindle assembly. **(A)** In WT oocytes, phospho-Aurora signals were enriched on centromeres and robust microtubule fibers radiated from opposite poles to form a bipolar spindle at metaphase I (left). During anaphase I (right), phospho-Aurora signals accumulated at the central spindle, and interchromosomal microtubules formed between the two masses of segregating homologous chromosomes. **(B)** *Aur1* KD caused a range of defects in meiotic spindle assembly at metaphase I. These defects were categorized, with proportions labeled in the merge panel (n = 315). (I) 6% showed normal spindles with lengths of 4–5 μm; (II) 2% showed elongated spindles with lengths >6 μm; (III) 4% showed compact spindles with lengths <4 μm, exhibiting delocalized Aurora signals surrounding chromosomes; (IV) 24% showed thick microtubules radiating from opposite poles to form rudimentary spindles; (V) 10% lacked spindles with few microtubules attached to chromosomes; and (VI) 42% lacked spindles and showed no detectable microtubule staining. In categories IV to VI, diffuse Aurora signals were spread along chromosomes. In addition, small proportions of defects in homolog chromosome congression were also observed and distributed in the following categories: (VII) 1% showed one homologous chromosome (arrowhead) detached from the spindle; (VIII) 2% showed one bivalent (a pair of homologous chromosomes, arrowhead) detached from the spindle; (IX) 3% showed two separate chromosomes (arrowhead) detached from the spindle, and the spindle seemed to be multi-polar; and (X) 6% showed three separate bivalents with microtubules between them. **(C)** Within 5 min after exposure to WT sperm, the progression of anaphase I in *Aur1* KD oocytes was analyzed (n = 245). (I) 93% showed defective spindles. Insert panel showed a sperm nucleus inside an *Aur1* KD oocyte. (II) 5% showed normal segregating chromosomes with enriched Aurora signals on the central spindle and less robust interchromosomal microtubule fibers. (III) 2% showed lagging chromosomes (arrowhead) along the central spindle. **(D)** In all *Aur2* KD oocytes (n = 26), chromosomes were decondensed, and phospho-Aurora signals and microtubule staining were absent. Scale bars: 2 µm.

The first meiotic spindle is organized without centrosomes in *O. dioica*, potentially through a chromatin-dependent pathway mainly driven by Aurora kinases. The enrichment of Aurora signals on centromeric regions was concomitant with the establishment of robust microtubules around the chromosomes (Figure 2A). Intriguingly, a variety of defects in meiotic spindle assembly in *Aur1* KD oocytes were observed and classified into 10 categories based on spindle morphology and karyotype (Figure 2B). Among 315 oocytes from six *Aur1* KD females, only 6% had normal-looking spindles. An additional 36% of spindles appeared to be elongated, compact, multi-polar, or rudimentary, with diffuse Aurora signals on most chromosomes. Spindles were absent in the remaining majority, with few, sporadic microtubules in contact with chromosomes. Interestingly, in 6% of oocytes, we also observed homologous chromosomes detached from a partially organized spindle. To assess the extent that these defects can affect chromosome segregation, *Aur1* KD oocytes were fertilized with WT sperm and collected within 5 min post-fertilization to analyze the progression of anaphase I. In WT, Aurora signals relocated to the central spindle and overlapped with fine and robust microtubule fibers which emerged between segregating homologous chromosomes, when thick microtubule fibers at the two poles were degraded (Figure 2A). In contrast, among 245 oocytes from six *Aur1* KD females, 93% of defective spindles were not capable of segregating chromosomes. In rare cases when Aurora signals were enriched on the central spindle and chromosomes managed to segregate, 5% showed less robust microtubule staining in the central spindle, and 2% showed lagging chromosomes along the central spindle (Figure 2C). In all the *Aur2* KD oocytes observed in seven females, neither Aurora signals nor microtubules were detected around decondensed chromosomes (Figure 2D).

In summary, Aurora1 was involved in the enrichment of Aurora2 on centromeres and the regulation of microtubule dynamics to promote acentrosomal spindle assembly that was particularly driven by centromeric Aurora2. The deregulation of Aurora1 can lead to defective chromosome congression during metaphase I and subsequent mis-segregation during anaphase I.

### INbox motif of INCENPa activates both Aurora1 and Aurora2

**FIGURE 3:**
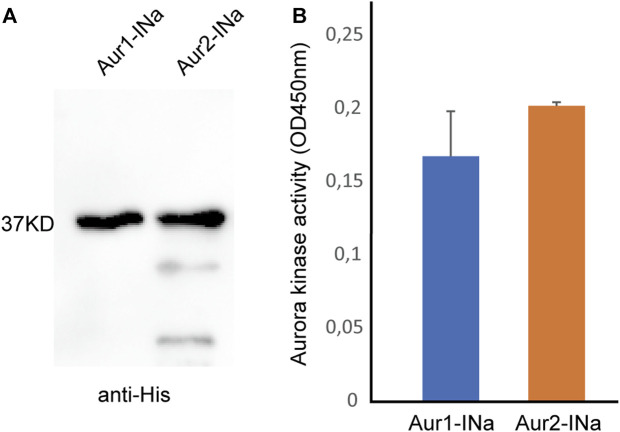
Kinase activity assay of Aurora1 or Aurora2 complexed with the INbox motif of INCENPa. **(A)** Western blot of purified Aurora1 and Aurora2 complexed with the INbox of INCENPa (INa) using an anti-His tag antibody. **(B)** INbox motif can stimulate the kinase activities of Aurora1 and Aurora2. Mean values with standard errors were calculated from four independent experiments.

It was observed that Aurora1 can assist Aurora2 in regulating meiotic progression. Then, we assessed whether Aurora1 has any intrinsic enzymatic activity. To make a comparison, the His-tagged full-length proteins of Aurora1 and Aurora2 were expressed and purified in complexes with the INbox motif of INCENPa using a bi-cistronic vector pHTvAMP1-SGC in *E. coli*. Western blot analyses confirmed that the concentrations of purified protein complexes were similar before carrying out the Aurora kinase assay (Figure 3A). Interestingly, *in vitro*, INCENPa activated Aurora1 to similar levels as it activated Aurora2 when provided with a synthetic substrate that contained an Aurora consensus phosphorylation site (Figure 3B). This implies that *O. dioica* Aurora1 is a *bona fide* Aurora kinase and can be activated by a known interaction partner.

### Novel interaction proteins of Aurora1 in *O. dioica*

Aurora kinases are organized in regulatory kinase cascades by their interaction/scaffolding proteins that are involved in the activation and spatiotemporal targeting of kinases. Our initial genome-wide search failed to detect TPX2, a microtubule-binding protein that normally localizes Aurora A toward the spindle and the mid-body (Bayliss et al., 2003), implying that the Aurora1-scaffold complement might have changed in *O. dioica*. Considering new interaction proteins of Aurora1, a cDNA prey library (number of independent clones: 9.60×10^6^) generated from oocytes and early embryos of *O. dioica* was screened with Aurora1 as bait using a yeast two-hybrid library screening approach. This approach identified eight possible interaction proteins (Table 1). To our knowledge, none of them have been previously reported as Aurora A interaction proteins.

**TABLE 1:** *O. dioica* Aurora1 interaction proteins identified by a yeast two-hybrid screen.

Protein name	NCBI accession no.	Expression profile	Function
c-Jun-amino-terminal kinase-interacting protein 4, JIP4	CBY17774.1	All stages	Scaffold protein mediating JNK signaling; spermatozoa–egg interaction; and lysosomal positioning
Nipped-B-like protein, Scc2/Nipbl	GSOIDP00013090001^a^	All stages	Forms Scc2/Scc4 heterodimeric complex that regulates cohesin loading
Mitochondrial carrier homolog 2	CBY22100.1	All stages	Mitochondria fusion
Tetratricopeptide repeat protein 36, Ttc36	CBY23270.1	Tail shift, day 2, testis	Proximal tubule marker in the mouse kidney cortex
Dmx-like protein 1	CBY20658.1	All stages	Contains a large number of WD repeats
Peroxidase-like	CBY07839.1	Somatic cells after the tail shift	Breaks down hydrogen peroxide
Calcium/calmodulin-dependent 3′,5′-cyclic nucleotide phosphodiesterase 1C	CBY23339.1	1hpf; hatched; early tadpole; tail shift; day 1, 2, and 5; and testis	Regulates the intracellular levels of second messengers cAMP and cGMP and controls Ca^2+^ homeostasis
Unnamed protein	CBY11821.1	All stages	—

^a^ OikoBase accession number.

## Discussion

### *O. dioica* Aurora1 might have rewired its interaction network

Given the loss of canonical interaction proteins of Aurora A, such as TPX2 in *O. dioica*, it is perhaps not surprising that several new binding partners of Aurora1 may have developed. c-Jun-amino-terminal kinase-interacting protein 4 (JIP4) is the scaffolding protein in the JNK/mitogen-activated protein kinases (MAPK) signaling pathway (Nguyen et al., 2005). JIP4 is involved in the bidirectional transportation of cargos along the microtubules through interaction with kinesin-1 and dynactin/dynein motors (Montagnac et al., 2009). The interface between JIP4 and kinesin-1 lies in the tetratricopeptide repeat (TPR) motif of kinesin-1 light chain (KLC1). Interestingly, we also identified a TPR-containing protein (Ttc36) as an Aurora1 interaction protein. Although Ttc36 is less studied, the TPR motif comprises 3–16 tandem-repeats of 34 amino acid residues and forms an all-helical structure, often mediating protein–protein interactions and cell cycle regulation (D’Andrea and Regan, 2003). Thus, it is tempting to speculate that JIP4 brings Aurora1 in close proximity to microtubule motors, allowing Aurora1 to phosphorylate kinesin-1 or dynactin/dynein subunits and regulate microtubule dynamics. Aurora A phosphorylates dynactin’s largest subunit p150^glued^ on its N-terminal microtubule-binding domain (MBD), which regulates the disassociation of the dynactin/dynein complex from the spindle poles after NEBD in *Drosophila* S2 cells (Romé et al., 2010). This phosphorylation is also involved in central spindle assembly at anaphase in human cells (Reboutier et al., 2013). It would be of great interest to test whether Aurora1 regulates microtubule dynamics through the interaction with JIP4.

Scc2/Nipbl regulates the loading of cohesin onto chromatin before the S phase (Mattingly et al., 2022). In addition to cohesin loading, Scc2 can move between cohesin complexes on chromosomes after DNA replication (Rhodes et al., 2017). Its interaction with Aurora1 suggests that Aurora1 might make transient contact with cohesin complexes at crossover sites, where homologous chromosomes are held together, and on centromeres between sister chromatids in meiosis I. Indeed, we observed one or two homologous chromosomes as being detached from the main chromosome mass in a minor proportion of *Aur1* KD oocytes. Further investigation is needed to test whether Aurora1 is involved in the maintenance of cohesion at metaphase I.

Although the function of Aurora in mitochondria is outside the scope of this study, we did identify mitochondrial carrier homolog 2 (MTCH2), a regulator of mitochondrial fusion, as an Aurora1 interaction protein (Bahat et al., 2018). Human Aurora A is transported into mitochondria in the interphase and induces mitochondrial fragmentation (Bertolin et al., 2018). This transport relies on its N-terminal 36 amino acids serving as an atypical mitochondrial targeting sequence (MTS). Once inside the mitochondria, MTS is cleaved off, generating two forms of truncated Aurora A. The N terminal of *O. dioica* Aurora1 is approximately 90 aa shorter than human Aurora A and does not have an MTS motif (Supplementary Data S1). It is possible that MTCH2 might serve as a transporter to import Aurora1 into the mitochondria in *O. dioica*.

It would be of interest to develop specific antibodies for the aforementioned interaction proteins, in order to assess these interactions *in vivo* by Co-IP and colocalization assays. Targeted perturbations would be informative to dissect their roles in the regulation of Aurora1 in more detail.

### Evolution of Aurora homologs in appendicularians

Considering their distinct roles in meiotic spindle organization, we argue that the two Aurora kinases co-exist in meiotic nuclei. Aurora1 has opportunities to contact microtubules that are synthesized through an Aurora2-dependent pathway on chromosomes. This proposed pattern is distinct from other invertebrate and vertebrate models reported thus far. Aurora1 in *O. dioica*, or in appendicularians in general, may have evolved to regulate microtubule dynamics during meiotic spindle assembly. The role of *O. dioica* Aurora2 in regulating chromosome condensation and acentrosomal spindle assembly is consistent with the roles in vertebrates. However, a key residue (Gly198 in human Aurora A or Asn142 in human Aurora B) in the catalytic domain that determines the interaction partners, subcellular localizations, and functions of the respective Aurora kinases in vertebrates has been altered to Gly in the two *O. dioica* Aurora kinases (Gly105 in Aurora1 or Gly91 in Aurora2, Supplementary Data S1) (Fu et al., 2009). The two Aurora kinases in *O. dioica* share this feature with the single ancestral Aurora which possesses Gly at the equivalent residue in most invertebrate deuterostomes, social amoeba, and yeast.

A question raised for many years is why independent evolution of Aurora kinases in a few invertebrates has led to a similar functional separation in spindle and chromosome regulations. Although our study cannot definitively respond to this question, we can provide some new insights. As shown in this study, *O. dioica* Aurora1 has substantially changed its interaction proteins and regulated meiotic spindle organization from distinct locations. There is also cross-talk between Aurora1 and Aurora2 in promoting their respective localizations, along with other signaling cascades that are brought together by novel interaction/scaffolding proteins.

## Data Availability

The datasets presented in this study can be found in online repositories. The names of the repository/repositories and accession number(s) can be found at: https://www.ncbi.nlm.nih.gov/genbank/, SCLG00000000.1 https://www.ncbi.nlm.nih.gov/genbank/, SDII00000000.1.
