# Supplementary material for: Functional specialization of Aurora kinase homologs during oogenic meiosis in the tunicate Oikopleura dioica
Source: Front Cell Dev Biol. 2023 Dec 7;11:1323378. doi: 10.3389/fcell.2023.1323378 (PMC10733467; doi:10.3389/fcell.2023.1323378)
Supplement: Supplementary file 1 [file Table1.DOCX]

***Supplementary Material***

FbAur1 MD---TENLKPKS---------------------SENT-----FQ--------ADQNEEK 23

OdAur1 ------------------------------------------------------------ 0

OaAur1 ------------------------------------------------------------ 0

FbAur2 ------------------------------------------------------------ 0

OdAur2 ------------------------------------------------------------ 0

OaAur2 ------------------------------------------------------------ 0

CiAur ------------------------------------------------------------ 0

PmAur ------------------------------------------------------------ 0

HsAurB --MAQKENSYPWP--------------------------YGR------QTAPSGLSTLPQ 26

HsAurC ------------------------------------------------------------ 0

XlAurB --MSYKENLNPSSY-------------------TSKFTTP-------------SSATAAQ 26

HsAurA MDRS-KENCISGPVKA-TAPVGGPKRVLVTQQFPCQNPLPVNSGQAQR---VLCPSNSSQ 55

ApAur MTPT-----GPSH----HSMA--PKRVL---PAASQSNMYGNIRSASTTSTTASTSSQAL 46

XlAurA MERAVKENHKPSNVKIFHPMTEGAKRIPVNQPQSTQFRPPGTAVSAQR---ILGPSNVPQ 57

SpAur -----------------------------------------------M---HVHLSLQ-- 8

BfAur ------------------------------------------------------------ 0

SkAur ----------------------------------MSSKKIGSVESGAM---ASKTDYGPK 23

FbAur1 KCLAKQ-ETKHLHKHVQN---------------------------------------SIG 43

OdAur1 -------------------------------------------MSTTE---------K-- 6

OaAur1 -------------------------------------------MSNANREAGLPHEPAIQ 17

FbAur2 ------------------------------------------------------------ 0

OdAur2 -------------------------------------------MQVENKE---------- 7

OaAur2 ---------------------------------------------MENKE---------- 5

CiAur -------------------------------------------MSSANNE---------- 7

PmAur ---------------------MNPPTVSS--STKMNSSSAKVPMGEVNKK---------- 27

HsAurB RVLRKEPVTP----------------------------SALVLMS--------------- 43

HsAurC ------MSSP----------------------------RAVVQLG--------------- 11

XlAurB RVLRKEPYVST-----------------------FTTPS-------DNL---LAQRTQLS 53

HsAurA RIPLQAQKLVSSHKPVQNQKQ------KQLQATSVPHPVSRPLNN----------TQKSK 99

ApAur RLLQNAKTSKNADNRIAHAERQGPPSAH---PAAMQKPAARVAPSNENRPDPAARQHQHQ 103

XlAurA RVLAQAQ------KPILS--SQKPTTQIPLRPATQGHQSSKPQGPNENRN-PQQTSHSS- 107

SpAur -VASSL----------------------------NRGTTA-------------QARTQP- 25

BfAur ------------------------------------------------------------ 0

SkAur RVLKET----------------------------NQQTSSKP----------EAKFAKP- 44

FbAur1 RSLPMQNS---SPIRRESDPLLLQHQTPNDPSITTPLNWKISDFDVGRLLGSGKFGRVYL 100

OdAur1 --IEQVSG--------DNKENEIDSCIPTKKSSGANKAWKISDFDMGKALGKGRFGHVYC 56

OaAur1 VTTTKVTA--------KVTDTEMRPPGGDNKENSNAKPWKIDDFQLGKPLGKGRFGNVYM 69

FbAur2 -----------------------MTPKGRTNTPNRGGDWQLKDFEIGRPLGKGKFGSVYL 37

OdAur2 ------------N-------------TDPSGSKETRDDWSLAKFDIGKPLGKGKFGSVYL 42

OaAur2 ------------NIP----VEDPKLENKQEPSPDSSEDWSLKRFDIGKPLGKGKFGSVYL 49

CiAur -------------NKAPEKASTSGRATASSAKDNASDTWSLKNFDIGKPLGRGKFGSVYL 54

PmAur -------------SQQEDKQ----PTKSGAQNKPQKKEWSLKNFDIGKPLGRGKFGSVYL 70

HsAurB ----------RSNVQPTAAPGQKVMENSSGTPDILTRHFTIDDFEIGRPLGKGKFGNVYL 93

HsAurC ------------KAQPA---GEESNQTAQQPSSPAMRRLTVDDFEIGRPLGKGKFGNVYL 56

XlAurB RITPSASSSVPGRVAVS----TEMPSQNTALAEMPKRKFTIDDFDIGRPLGKGKFGNVYL 109

HsAurA QPLPSAPENN----------PEEELASKQKNEESKKRQWALEDFEIGRPLGKGKFGNVYL 149

ApAur QQLQQQKATGHDRVLKESQAGNSTTTTMTSTQSKEANKWSLANFDIGRPLGKGKFGNVYL 163

XlAurA --TPNVEKKG---------STDQGKTSAVPKEEGKKKQWCLEDFEIGRPLGKGKFGNVYL 156

SpAur --VKSA---------------ENG-HGHDDKQMEPKKSWTLKDFDIGRPLGKGKFGSVYL 67

BfAur -----------------------------------MKSWKLEDFDIGRPLGKGKFGNVYL 25

SkAur --LPQN---------------EPA-Q---GPGNHTRKKWTLSDFDIGRPLGKGKFGNVLL 83

: *::*: ** *:** *

FbAur1 AREK-------SKKTIIAIKSIKKEDIRKEKIKYQLVREIEIQKNLHHFNILKMWGYFYD 153

OdAur1 ARET-------KSGYVVALKIMFKNQIKDANLQHQVRREVEIQSHIKHKNICRLYGYFHD 109

OaAur1 AKEK-------KSNYIVALKVLFKSQIREAGLQHQVRREVEIQSHIKHKNICRLYGYFHD 122

FbAur2 ARTGPKKNKPNEPPMICAIKVLFKSQLQKYGVEHQLRREIEVQFNVKHEYVLPMYGYFWD 97

OdAur2 ARTK-------KEKYIVAVKILFKSQLVTGGVEAQLRREIEIQSHLRHPHILRLFGWFHD 95

OaAur2 AREK-------KSKFIIALKLLFKSQLVSNNVENQLRREIEIQSHMRHPHILRLFGWFHD 102

CiAur AREK-------KSKFIVALKVLFKSQLMKSNVEHQLRREIEIQSHLRHPHILRLYGYFHD 107

PmAur ARER-------ESKFIVALKVLFKSQLVKSNVEHQLRREIEIQSHLRHPHILRLYGYFHD 123

HsAurB AREK-------KSHFIVALKVLFKSQIEKEGVEHQLRREIEIQAHLHHPNILRLYNYFYD 146

HsAurC ARLK-------ESHFIVALKVLFKSQIEKEGLEHQLRREIEIQAHLQHPNILRLYNYFHD 109

XlAurB AREK-------QNKFIMALKVLFKSQLEKEGVEHQLRREIEIQSHLRHPNILRMYNYFHD 162

HsAurA AREK-------QSKFILALKVLFKAQLEKAGVEHQLRREVEIQSHLRHPNILRLYGYFHD 202

ApAur AREK-------KSKFIVALKVLFKSQLQKAKVEHQLRREIEIQSHLRHDHILRLYGYFYD 216

XlAurA ARER-------ESKFILALKVLFKSQLEKAGVEHQLRREVEIQSHLRHPNILRLYGYFHD 209

SpAur AREK-------QTKYIVALKVLFKSQLQKAQVEHQLRREIEIQSHLRHPNILRLFGYFYD 120

BfAur AREK-------NSKFIVALKVLFKSQLMKAGVEHQLRREIEIQSHLRHPHILRLYGYFYD 78

SkAur AREK-------KSKFILALKVLFKSQLQKAGVEHQLRREIEIQSHLRHPNILRMYGYFYD 136

*: . : *:* : * :: :: *: **:*:* :::* : ::.:* *

FbAur1 AKRVYILLEYAPEGALWRVLK--LCGRLPNILCATYCYQMIQCLKYLHSLKIIHRDIKPE 211

OdAur1 DRRVYIILEFCKNGNLFTKLK--EVKKFESIEAARYVREIAEGLDYIHKLNVIHRDLKPE 167

OaAur1 TKRIFIILEFCKNGNLYTKLK--EQMRMTSKDSARYVQEIAEGLHHIHKLNVIHRDLKPE 180

FbAur2 AKRVFLVLEFASGGELYANMQ--KKGTFSQTETATYIYEICEALKVCHANDVWHRDIKPE 155

OdAur2 VKKIYLVLEYAAQGELYKELM--KKGRLSEFRTATIIHEVSDAMKYCHANKIIHRDLKPE 153

OaAur2 SKKIYLILEFAGKGELYKELM--KEGRLSEFRASTIIYEVADALNYCHANNIIHRDIKPE 160

CiAur ETRVYLILEYASRGEMYKELQ--KQGKFTEEMSATYIAELADALNYCHSKQVIHRDIKPE 165

PmAur ETRVYLILEYAPRGEMFKELM--KQGRFNEELTATYIAELADALKYCHSKKVIHRDIKPE 181

HsAurB RRRIYLILEYAPRGELYKELQ--KSCTFDEQRTATIMEELADALMYCHGKKVIHRDIKPE 204

HsAurC ARRVYLILEYAPRGELYKELQ--KSEKLDEQRTATIIEELADALTYCHDKKVIHRDIKPE 167

XlAurB RKRIYLMLEFAPRGELYKELQ--KHGRFDEQRSATFMEELADALHYCHERKVIHRDIKPE 220

HsAurA ATRVYLILEYAPLGTVYRELQ--KLSKFDEQRTATYITELANALSYCHSKRVIHRDIKPE 260

ApAur DTRVYLILEYAARGELYKEMQAQKAGHFDEDRSAVYIYQLAKALLYCHEKKVIHRDIKPE 276

XlAurA ASRVYLILDYAPGGELFRELQ--KCTRFDDQRSAMYIKQLAEALLYCHSKKVIHRDIKPE 267

SpAur ESRVYLILEYAPRGELYKQLQ--RAGRFDEQRTASYISQLADALKYCHSKKVIHRDIKPE 178

BfAur DTRVYLILEYAPRGELYKELQ--KQVRFDERRSATYIAQLADALKYCHSKKVIHRDIKPE 136

SkAur DIRVYLILEFAPQGELYKELQ--KRGRFDEKRSATYVGQLADALKYCHAKKVIHRDIKPE 194

:::::*::. * :: : : . : :: . : * : ***:***

FbAur1 NCLVGCPTEIT------------------------------------------------- 222

OdAur1 NVLLGRNNEVKLADFGWCVFTPQSRRQTFCGTMDYLSPEMLNGVSHDKKIDHWALGCIAF 227

OaAur1 NVLLGKNHEVKLADFGX------------------------------------------- 197

FbAur2 NILIGYHGELKLADFGWSVHGTG-KRQTMCGTPDYLPPEILSGQDYGPAVDMWAVGVLNY 214

OdAur2 NVLIGLQGEAKLADFGWSVRTPSRRRETMCGTLDYLPPEMVEQVDYTFTVDNWTIGVLCY 213

OaAur2 NILVGLQGEVKLADFGWSVRTPSKRRATMCGTLDYLPPEMVEQKDYDKKVDNWTVGVLCY 220

CiAur NLLMGLRGELKIADFGWSVHAPSSKRQTLCGTLDYLPPEMIEAKDHDANVDLWTLGILCY 225

PmAur NLLMGLRGELKIADFGWSVHAPSSKRQTLCGTLDYLPPEMVEGKNHDEGVDLWTLGILCY 241

HsAurB NLLLGLKGELKIADFGWSVHAPSLRRKTMCGTLDYLPPEMIEGRMHNEKVDLWCIGVLCY 264

HsAurC NLLLGFRGEVKIADFGWSVHTPSLRRKTMCGTLDYLPPEMIEGRTYDEKVDLWCIGVLCY 227

XlAurB NLLMGYKGELKIADFGWSVHAPSLRRRTMCGTLDYLPPEMIEGKTHDEKVDLWCAGVLCY 280

HsAurA NLLLGSAGELKIADFGWSVHAPSSRRTTLCGTLDYLPPEMIEGRMHDEKVDLWSLGVLCY 320

ApAur NLLLDLKGDLKIADFGWSVHAPSSRRATLCGTLDYLPPEMIEGKTHDEKVDLWSLGVLCY 336

XlAurA NLLLGSNGELKIADFGWSVHAPSSRRTTLCGTLDYLPPEMIEGRMHDETVDLWSLGVLCY 327

SpAur NLLLGLLGDLKIADFGWSVHAPSSRRNTLCGTMDYLPPEMIEGRMHDDKVDLWSLGVLCY 238

BfAur NLLLGLKGDLKIADFGWSVHAPSSRRATLCGTLDYLPPEMIEGKMHDEKVDLWSLGVLCY 196

SkAur NLLLGLRGDLKIADFGWSVHAPSSRRATLCGTLDYLPPEMIEGRMHDEKVDLWSLGVLCY 254

* *:. : .

FbAur1 ------------------------------------------------------------ 222

OdAur1 ELLTGYPPFVGDKKNPTVEVTRSLIVSGTIDFEKAKEVNEQEQEVVDALVQLEPNDRIEL 287

OaAur1 ------------------------------------------------------------ 197

FbAur2 ELLVGNPPFEEDNMEETYRRI------KQGIYQFPQNVSQLAKDCIRRMLVVNAERRAKP 268

OdAur2 ELLTGKPPFEHDDKNVTYQRI------VNTQFTYPNHVKEGARDLITRLLQYKGANRIPL 267

OaAur2 ELLCGKPPFETESNADTYKRI------VNTEYTFPDVIREDAX----------------- 257

CiAur EFLVGKPPFETKSTQETYLRI------TSLKYSFPPHVSEGARDLIRRLLKLEPRHRLPL 279

PmAur EFLVGKPPFESQSNQDTYKRI------TQLQYTFPPHVSDGAKDLIRKLLQRNPRHRLPL 295

HsAurB ELLVGNPPFESASHNETYRRI------VKVDLKFPASVPMGAQDLISKLLRHNPSERLPL 318

HsAurC ELLVGYPPFESASHSETYRRI------LKVDVRFPLSMPLGARDLISRLLRYQPLERLPL 281

XlAurB EFLVGMPPFDSPSHTETHRRI------VNVDLKFPPFLSDGSKDLISKLLRYHPPQRLPL 334

HsAurA EFLVGKPPFEANTYQETYKRI------SRVEFTFPDFVTEGARDLISRLLKHNPSQRPML 374

ApAur EFLVGKPPFESQGNTETYRKI------TKVEFTFPKHVSEGARDLICKLLKHNPSHRLSL 390

XlAurA EFLVGKPPFETDTHQETYRRI------SKVEFQYPPYVSEEARDLVSKLLKHNPNHRLPL 381

SpAur EFLVGKPPFEAEGSTETYRRI------TKVHYQFPSYVSAGARDVIKRLLQHNPANRLPL 292

BfAur EFLVGKPPFEAEGHSETYRRI------SKVDLRFPPHVTSGARDLISKLLRHNPMLRLPL 250

SkAur EFLVGKPPFEAEGHSETYRKI------SKVDFRYPSFVTDGARDLVSKLLRHNPAMRLSL 308

FbAur1 ---------------------------------------- 222

OdAur1 VELLKMRWLAE----------------------------- 298

OaAur1 ---------------------------------------- 197

FbAur2 VQIQTHSWIMKYARPHIFKNNKYVGRINLVSKKIEQVAQE 308

OdAur2 DQLQRHAWIREHAVPHRWTEDHFPVPGYPNYSD------- 300

OaAur2 ---------------------------------------- 257

CiAur ESVMAHPWIKANAKVHKFGPDGRPMD-------------- 305

PmAur DDVMAHEWIKANAKVHKFGPDGRPLY-------------- 321

HsAurB AQVSAHPWVRANSRRVLPPSALQSVA-------------- 344

HsAurC AQILKHPWVQAHSRRVLPPCAQMAS--------------- 306

XlAurB KGVMEHPWVKANSRRVLPPVYQSTQSK------------- 361

HsAurA REVLEHPWITANSSKPSNCQNKESASKQS----------- 403

ApAur EGVIAHAWIQEKISQRS----------------------- 407

XlAurA KGVLEHPWIIKNSQLKKKDEPLPGAQ-------------- 407

SpAur EQVLAHPWIVENSKKKPSSSSTSSSESQS----------- 321

BfAur DSVLSHPWIKDNAATQAESTPAHPTHASHTKS-------- 282

SkAur DGVLNHPWIKEHGVLSEPTSSSSSS--------------- 333

**Supplementary Data S1**. Multiple sequence alignment of Aurora kinases in three larvaceans and representative deuterostomes. The C-terminal regions of Aurora1 in *O*. *albicans* and *F*. *borealis* are missing in the larvacean transcriptome data. Thus, the aligned sequences in part of the catalytic domain (indicated by black lines at the top) are used in phylogenetic analysis. The critical residues (Gly198 in human Aurora A and Asn142 in human Aurora B) that determine the interaction partners and localizations of Aurora kinases are shaded in grey. The N-terminal mitochondrial targeting sequence (MTS) in human Aurora A is highlighted in yellow. The abbreviations of selected species are: Hs, *Homo sapiens*; Xl, *Xenopus laevis*; Od, *Oikopleura dioica*; Oa, *Oikopleura albicans*; Fb, *Fritillaria borealis*; Ci, *Ciona intestinalis*; Pm, *Phallusia mammillata*; Bf, *Branchiostoma floridae*; Sk, *Saccoglossus kowalevskii*; Ap, *Asterina pectinifera*; Sp, *Strongylocentrotus purpuratus*. The symbols under the alignment indicate degrees of identity: *(identical)>:(strong similarity)>.(weak similarity).


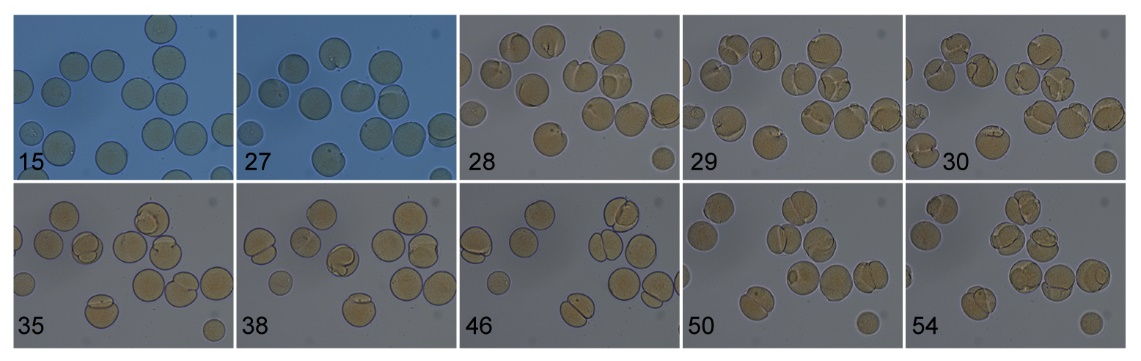


Supplementary Figure S1. Representative time-lapse images of *Aur1* KD oocytes after exposure to wild type sperm. Time (minutes) post fertilization was shown at the bottom left corner.
